# Pedophilic Disorder and Suicide Risk: Understanding the Intersection

**DOI:** 10.1192/j.eurpsy.2025.2299

**Published:** 2025-08-26

**Authors:** B. V. Ferreira, C. Alves, J. F. Fontes, M. M. Serra, T. Pereira, R. R. Henriques, M. Gonçalves

**Affiliations:** 1 Clínica 5; 2 Clínica 3; 3 Clínica 2, ULS São José - Hospital Júlio de Matos, Lisboa, Portugal

## Abstract

**Introduction:**

Pedophilic disorder is characterised by a sustained, focused, and intense pattern of sexual arousal involving pre-pubertal children. In addition, the individual must have acted on these thoughts, fantasies or urges or be markedly distressed by them (ICD - 11).

Beyond its obvious ethical and legal implications, this disorder is often associated with substantial mental health comorbidities, including depression, anxiety, and substance abuse. One particularly alarming yet understudied aspect is the elevated risk of suicidal ideation and behavior among individuals diagnosed with pedophilic disorder. The intersection between pedophilic disorder and suicide risk is critical to understand, as it can inform the development of more effective intervention strategies and support systems.

**Objectives:**

The present review aims to explore the intersection between pedophilic disorder and suicide risk, shedding light on the psychological factors that contribute to suicidal ideation and behavior among individuals with this disorder, including those who have never sexually engaged with children.

**Methods:**

PubMed, Web of Science and Google Scholar were searched using the following keywords “pedophilic disorder”, “suicide risk”, “suicidal ideation”, and “stigma”. Articles published within the last decade were included.

**Results:**

The review showed several risk factors, perhaps the most significant being the psychosocial effects of the intense stigma, social isolation, and internalized shame. Other risk factors are prior mental health treatment, weaker attraction to adult women, history of sexual abuse, young age, less education, and additionally, the lack of access to appropriate mental health services.

The relationship between these individuals’ emotional distress and their likelihood of sexually engaging with a child is not known, but there is evidence that impulse control is compromised by negative affect. Moreover, literature posits that stigma raises the risk of sexual activity with children via disturbances in emotional, social, cognitive, and health service utilization domains.

**Conclusions:**

It is evident that this population faces unique challenges that require specialized attention and addressing this intersection necessitates a comprehensive approach.

Strategies aimed at reducing stigma, promoting social support, enhancing access to mental health services and fostering supportive environments have the potential to mitigate suicidal risk factors and enhance overall well-being. Furthermore, efforts to destigmatize help-seeking behaviors and improve access to confidential and non-judgmental support services are crucial in reducing the incidence of suicide and promote mental health among this population.

**Disclosure of Interest:**

None Declared

